# Developmental consequences of short apneas and periodic breathing in preterm infants

**DOI:** 10.1038/s41372-023-01748-8

**Published:** 2023-08-09

**Authors:** Alicia K. Yee, Leon S. Siriwardhana, Gillian M. Nixson, Lisa M. Walter, Flora Y. Wong, Rosemary S. C. Horne

**Affiliations:** 1 Department of Paediatrics, Monash University, Melbourne, VIC Australia; 2 Melbourne Children’s Sleep Centre, Monash Children’s Hospital, Melbourne, VIC Australia; 3 Monash Newborn, Monash Children’s Hospital, Melbourne, VIC Australia

**Keywords:** Outcomes research, Medical research

## Abstract

**Objective:**

We investigated the relationship between respiratory events experienced before and after hospital discharge and developmental outcomes at 6 months corrected age (CA).

**Study design:**

Preterm infants born between 28–32 weeks gestational age (GA) were studied at 32–36 weeks postmenstrual age (PMA), 36–40 weeks PMA, 3- and 6-months CA. Percentage total sleep time (%TST) with respiratory events (isolated apneas, sequential apneas and periodic breathing (PB)) at each study was calculated. Stepwise multiple linear regressions determined significant predictors of developmental outcomes at 6 months.

**Result:**

%TST with respiratory events at term were significant predictors of language (*R*^2^ = 0.165, β = −0.416) and motor (*R*^2^ = 0.180, β = −0.485) composite scores of the Bayley Scales of Infant Development at 6 months, independent of GA, birth weight and sex.

**Conclusions:**

In clinically stable very preterm infants at term equivalent age, time spent having respiratory events, was related to a reduction in language and motor outcomes at 6 months.

## Introduction

Preterm birth remains a problem worldwide as 1 in 10 infants are delivered prematurely annually [1]. While mortality rates have improved with improvements in neonatal and perinatal medicine, very preterm infants (born at 28–32 weeks gestational age (GA)) are still at twice the risk for neurodevelopmental impairments at 2 years of age [2]. Infants born preterm frequently have immature respiratory control which manifests as apnea. Apnea of prematurity (AOP) is one of the most common diagnoses in the neonatal unit and is defined as a cessation of breathing for ≥20 s or a shorter pause accompanied by bradycardia ( < 100 beats per minute), cyanosis, or pallor [3]. The prolonged apneas of AOP have been associated with adverse neurodevelopmental outcomes in preterm infants at 13 months [4] and 3 years of age [5]. AOP is commonly resolved by term age [6, 7]. However, preterm infants also have frequent shorter apneas (3–5 s in duration), which can occur in isolation or in repetitive patterns, that may not be detected clinically due to the current averaging times of oximeters used in the neonatal unit [8]. Studies have shown that short apneas (both isolated and clustered as seen in periodic breathing) are associated with falls in peripheral and cerebral oxygenation [9–12] and these continue for 6 months post term corrected age (CA) in many preterm infants [10–12]. Hypoxia during critical periods of brain formation is associated with detrimental effects on cognitive function, brain adaptive potential and plasticity [13]. In animal studies, rat pups exposed to mild intermittent hypoxia in a pattern similar to that experienced by preterm infants during periodic breathing showed alterations in brain structure and metabolism, as well as systemic and brain inflammation [14] and with permanent neurofunctional deficit and white matter hypomyelination [15]. These studies suggest that intermittent hypoxia, as a result of respiratory instability, has the potential to contribute to adverse neurodevelopmental outcomes in preterm infants.

Although prolonged apneas have been associated with adverse neurodevelopmental outcomes, the developmental impact of shorter apneas that are often undetected in the nursery have not been investigated despite studies showing that they are associated with falls in cerebral oxygenation. As temperament in preterm infants has been associated with neurodevelopment outcomes [16], we also included an assessment of infant temperament. In the current study we investigated the relationship between respiratory instability experienced before and after hospital discharge up to 6 months CA and developmental and behavioral outcomes at 6 months CA. We hypothesized that increased time spent with respiratory events would be associated with poorer short-term developmental and behavioral outcomes in clinically stable very preterm infants.

## Methods

### Subjects

Infants born between 28–32 weeks of GA were recruited between March 2018 and July 2021. We have specifically chosen this age group to minimize the complications of extreme prematurity. We did not recruit infants who continued to require ventilatory support or oxygen therapy, to avoid the confounding effect of parenchymal lung pathologies and chronic lung disease. Infants were not recruited if they had intrauterine growth restriction, a major congenital abnormality, major intracranial abnormality, or significant intraventricular hemorrhage (Grade III or IV), or if they had a hemodynamically significant patent ductus arteriosus because of the known independent effects on neurodevelopment. Ethical approval for this project was granted by Monash Health and Monash University Human Research Ethics Committees. Parents gave written informed consent before the first study. Results of the effects of periodic breathing on cerebral oxygenation in this cohort of infants have been published previously [12].

### Study protocol

Infants were studied longitudinally on 4 occasions: at 32–36 weeks postmenstrual age (PMA) whilst in Monash Newborn, at 36–40 weeks PMA in the Melbourne Children’s Sleep Centre if they had been discharged home or in the special care nursery if they had not been discharged; at 3- and 6-months post term CA, in the Sleep Centre or in their own home depending on COVID-19 pandemic restrictions in 2020–2022. At each of the 4 sleep studies, physiological recordings during 2–3 h of daytime sleep in the supine position were made. Physiological recordings included electrocardiogram, thoracic and abdominal breathing movements (Resp-ez Piezo-electric sensor, EPM Systems, Midlothian, VA, USA), airflow and nasal pressure measured using nasal cannula (Parker Healthcare Pty Ltd, Melbourne, Australia), and peripheral arterial oxygen saturation (SpO_2_) measured using an oximeter set with a 2 s averaging time (Masimo Radical 7 pulse oximeter, Masimo Corporation, Irvine, California, United States). Cerebral tissue oxygenation index (TOI, %) was measured using Near Infrared Spectroscopy (NIRO 200, Hamamatsu Photonics K.K., Hamamatsu City, Japan) [10, 17, 18]. Sleep state was scored in real time as active sleep (AS), indeterminate sleep (IS) or quiet sleep (QS) using established bedside behavioral criteria [19].

At 6 months CA, infants underwent developmental assessments by a trained neuropsychologist, blinded to the sleep study results. Developmental assessments were carried out using the Bayley Scales of Infant Development III (BSID-III), which is an assessment tool for determining developmental delays in children. The scales are adjusted for prematurity and assess five key developmental domains: cognition, language, motor, social emotional and adaptive behavior [20]. The normal reference index for each BSID-III scale is a mean composite score of 100 ± 15 standard deviation (SD) and values below 85 considered as indicating neurodevelopmental impairment [21]. Additionally, parents also completed the Infant Behavior Questionnaire – Revised (IBQ-R) Short Form. The IBQ-R assesses temperament in infants aged between 3 and 12 months. The IBQ-R contains 91 questions which are grouped into 14 scales that assess the following dimensions of temperament: activity level, distress to limitations, approach, fear, duration of orienting, smiling and laughter, vocal reactivity, sadness, perceptual sensitivity, high intensity pleasure, low intensity pleasure, cuddliness, soothability and falling reactivity. Activity level includes limb movements, squirming and locomotor activity; distress to limitations includes any form of distress involved in daily activities or when an infant is unable to perform a desired action; approach includes positive anticipation; fear includes sudden changes or inhibited approach due to a change in stimulation; duration of orienting includes interaction with a single object for a prolonged period of time, smiling and laughter during caregiving and play situations; vocal reactivity includes vocalization during daily activities; sadness includes generally lowered mood and activity; perceptual sensitivity includes detection of slight external stimuli; high/low intensity pleasure describe the amount of pleasure displayed related to high or low intensity stimuli; cuddliness includes reaction to being held by caregivers; soothability includes reduction of uneasiness or distress when soothed by a caregiver; and falling reactivity includes rate of recovery from distress or excitement [22]. Caregivers were asked to indicate on a 7-point Likert scale (1 = Never, 2 = Very rarely, 3 = Less than half the time, 4 = About half the time, 5 = More than half the time, 6 = Almost always, 7 = Always) on how often they observed each behavior in their infant in the previous week.

### Respiratory data analysis

Sleep and respiratory data were transferred via European Data Format to LabChart software (ADInstruments, Sydney, Australia) for analysis. Sleep state was analyzed in 30 s epochs as AS or QS; there were few epochs of IS and so these were included with AS. Three types of respiratory events were identified using visual examination: isolated apneas, defined as respiratory cessation (central or obstructive) lasting ≥3 s [23]; sequential apneas, defined as 2 sequential central apneas separated by normal breathing lasting ≤20 s and periodic breathing, defined as 3 or more sequential central apneas lasting ≥3 s interrupted by normal breathing lasting ≤20 s [24]. Duration of each isolated apnea was calculated from the start of the respiratory pause until the end and durations of sequential apneas and periodic breathing were measured from the beginning of first apnea until the end of the last apnea. The mean duration of % total sleep time (%TST) spent in each event type and with all events combined was calculated for each infant at each study. Measurements of heart rate (HR), peripheral SpO_2_ and cerebral TOI were calculated beat-to-beat during each respiratory event which was free of movement artifact during a baseline period (10 s prior to each event), during the event itself and during a 15 s post event period, which was included to account for the physiological delay and recording equipment processing delay in oxygen desaturation following the events [10, 17]. Due to the cyclical nature of the changes in HR, SpO_2_ and TOI that occur during repetitive apneas, percentage changes from baseline averaged over each respiratory event would not accurately reflect the repetitive falls in these parameters. Hence, the nadir in HR, SpO_2_ and TOI for each respiratory event was used to calculate maximal % change from baseline termed as the nadir % change, which was averaged for each infant at each study [10, 17]. Time spent with SpO_2_ < 90% and TOI < 55% during respiratory events were calculated at each study. These cut-offs were chosen as the SpO_2_ target in our neonatal unit is >90% [25], and an SpO_2_ of 85–89% has been associated with increased mortality and morbidity [26]. Cerebral oxygenation <55% has been associated with poor neurocognitive outcomes in preterm infants [27].

### Statistical analysis

Statistical analysis was performed using SPSS software v27 (IBM SPSS, Chicago, USA). Data were first tested for normality and equal variance using the Shapiro-Wilk test. To assess the effects of sleep state on the %TST spent with respiratory events, nadir % changes in HR, SpO_2_ and TOI, Mann-Whitney U tests were performed at each age. As no sleep state differences were identified, the data were combined. Due to missing values at Studies 2 (*n* = 2) and 3 (*n* = 7) as a result of COVID-19 restrictions in Melbourne which prevented infants being studied, statistical tests accounting for missing values were performed. To compare the effects of PMA on %TSTevents, nadir % changes in HR, SpO_2_ and TOI and time spent with SpO_2_ < 90% and TOI < 55%, linear mixed modeling was carried out with PMA as a fixed factor and infant as a random factor, co-varying for GA, birth weight and sex, followed by Bonferroni post hoc tests. Stepwise multiple linear regressions were performed to determine significant predictors of each of the 5 domains assessed by the BSID-III and 14 scales of the IBQ-R outcomes at 6 months CA with %TST of each respiratory event (isolated, sequential apneas and periodic breathing) and combined (%TSTevents) at each age, with GA, birth weight and sex included as independent variables. To account for missing data at studies 2 (*n* = 2) and 3 (*n* = 7), multiple imputations for %TSTevents were carried out prior to stepwise multiple linear regression analyses. Values are presented as median [interquartile range, IQR] if the distribution was not normal and mean ± standard deviation (SD) if distribution was normal. Statistical significance was taken at *p* < 0.05.

## Results

Forty infants were recruited; and underwent the first study whilst in Monash Newborn. Thirteen infants were lost to follow-up after they were discharged home as parents decided against continuing participation and one was lost to follow-up due to COVID-19 restrictions. Six infants were unable to be studied at Study 3 and 6 studies at 6 months CA were delayed ( ~ 1 month) due to COVID-19 restrictions in Melbourne which prevented us entering the infant’s home. The 26 infants who completed the sleep study and neurodevelopmental assessments at 6 months CA were included in this study. The 26 infants included in this study (17 female and 9 male) had a median [IQR] GA at birth of 30 [30, 31] weeks with a median birth weight of 1.4 [1.3, 1.6] kg, birth length of 41 [39, 44] cm and head circumference of 28 [27, 29] cm. Infant APGAR scores ranged from 1–9 (median 7) at 1 min, and 4–9 (median 9) at 5 min. All infants were administered caffeine after birth and 35% were still on caffeine treatment at the time of Study 1 (32–36 weeks PMA). Infants still on caffeine at the time of the first study had a median caffeine dose of 8.6 [7.8, 9.8] mg/kg calculated using the weight of infants at the first study and the dose given at the time of the study. None of the infants were discharged home on caffeine.

All 26 infants were studied at 32–36 weeks PMA, 24 infants at 36–40 weeks, 19 at 3 months CA and 26 at 6 months CA. Demographic and sleep characteristics of the infants at each study are presented in Table 1. All infants completed at least 3 of the 4 sleep studies. Neurocognitive assessments for 11 infants were delayed due to the closure of the clinic conducting the assessments until March 2022. However, the BSID-III outcomes were all adjusted for age.

**Table 1:** Infant demographics, total study time and total sleep time at 32–36 weeks PMA, 36–40 weeks PMA, 3- and 6-months CA. Data presented as median [IQR] or *N* (%).

	32–36 weeks PMA	36–40 weeks PMA	3 months CA	6 months CA
N infants	26	24	19	26
PMA (weeks)	34 [33, 35]	39 [38, 40]	54 [53, 54]	68 [67, 69]
Sex (% female)	25 (63)	17 (63)	12 (60)	17 (65)
Weight (kg)	1.9 [1.6, 2.1]	3.0 [2.5, 3.4]	6.1 [5.3, 7.0]	7.8 [6.6, 8.4]
Length (cm)	43 [41, 45]	46 [44, 48]	58 [56, 62]	66 [64, 68]
Head circumference (cm)	31 [30, 32]	34 [33, 35]	41 [40, 42]	44 [43, 45]
Total study time (min)	186 [168, 202]	174 [157, 226]	142 [116, 198]	115 [71, 143]
Total sleep time (min)	176 [153, 187]	156 [111, 185]	122 [92, 146]	72 [35, 91]

*PMA* postmenstrual age, *CA* corrected age.

### Respiratory events

**Fig. 1:**
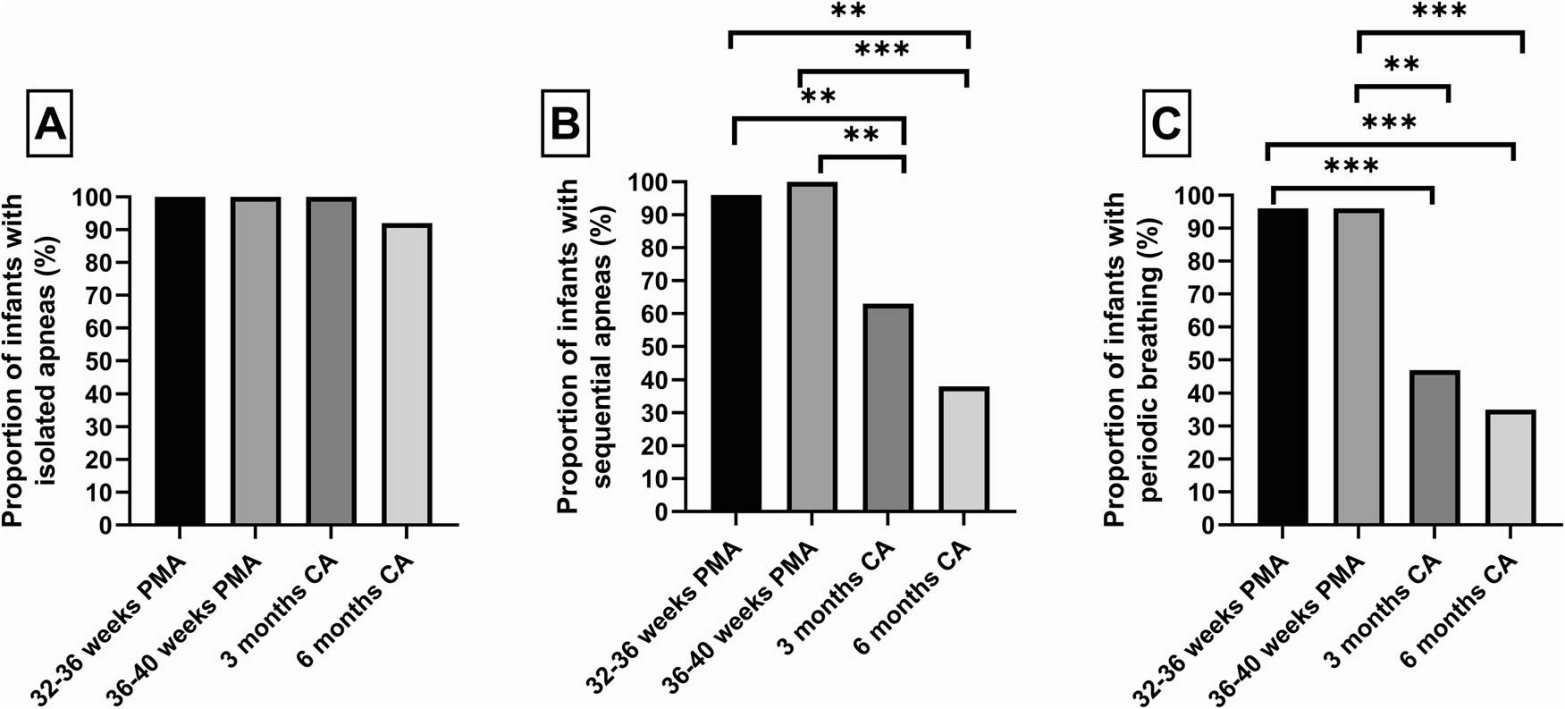
Proportion of infants having respiratory events at each study. Isolated apneas (**A**), sequential apneas (**B**) and periodic breathing (**C**) at 32–36 weeks PMA, 36–40 weeks PMA, 3 months and 6 months CA. PMA postmenstrual age, CA corrected age. **p < 0.01, ***p < 0.001.

**Fig. 2:**
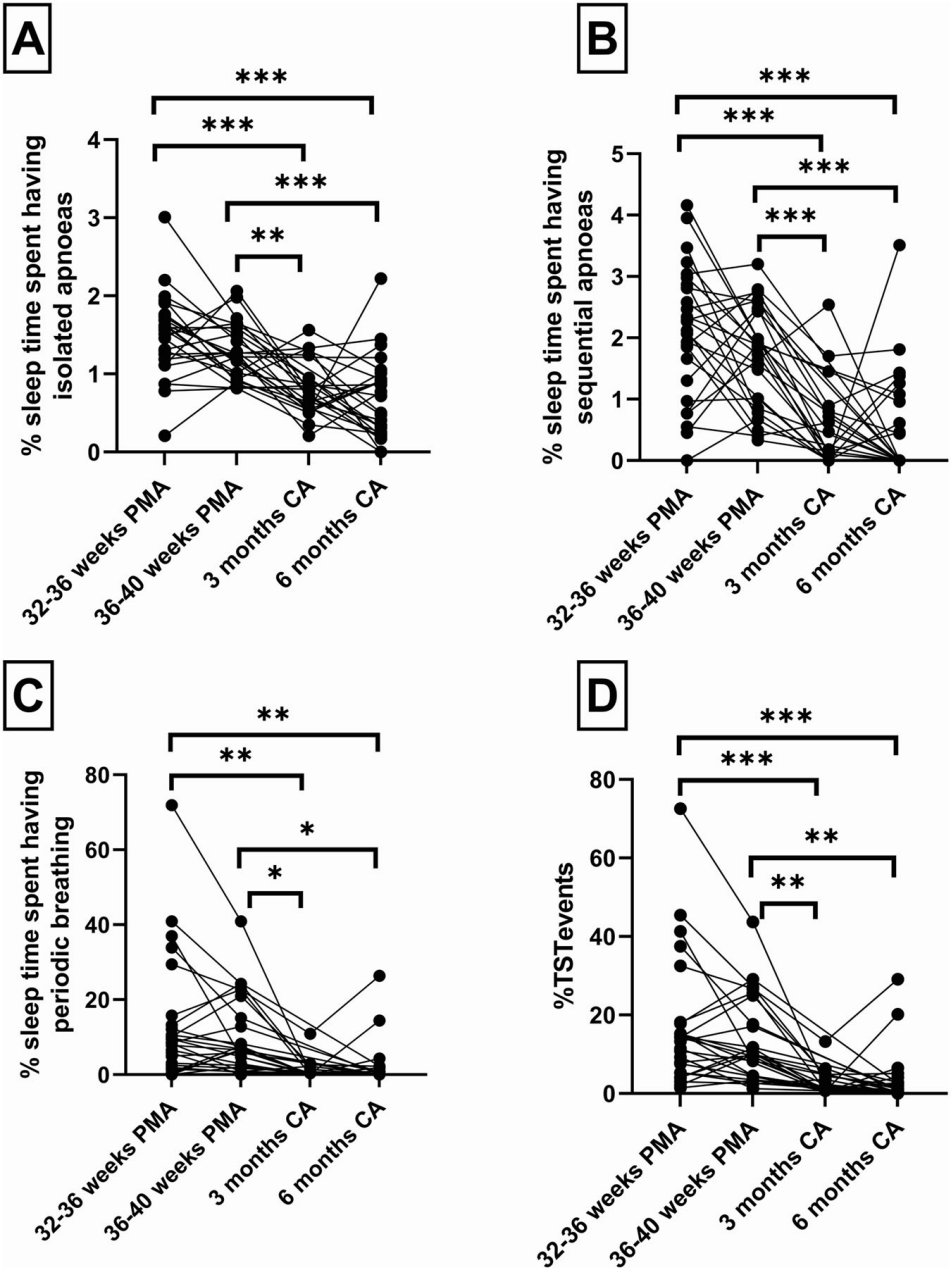
Percentage sleep time spent in respiratory events at each study. Isolated apneas (**A**), sequential apneas (**B**), periodic breathing (**C**) and combined respiratory events (**D**) for each infant at 32–36 weeks PMA, 36–40 weeks PMA, 3- and 6-months CA. PMA postmenstrual age, CA corrected age. *p < 0.05, **p < 0.01, ***p < 0.001.

All infants experienced isolated apneas up to 3 months CA and this fell to 92% at 6 months CA (Fig. 1A). In contrast, sequential apneas (Fig. 1B) and periodic breathing (Fig. 1C) were reduced to 63% and 47% respectively at 3 months and below 40% at 6 months. Figure 2 shows the %TST spent in each type of respiratory event. Periodic breathing occupied a median [IQR] of 8.5 [2.3, 13.8] % of TST at 32–36 weeks PMA and 6.8 [2.6, 19.5] % at 36–40 weeks PMA (Fig. 2C), while isolated apneas (Fig. 2A) and sequential apneas (Fig. 2B) occupied <5% TST at all ages.

### Physiological consequences of respiratory events

Of the 3227 respiratory events identified, 3079 events (95%) were free of artifact and were included in this part of the analysis: 1397 events (94%) at 32–36 weeks PMA, 1113 (97%) at 36–40 weeks PMA, 302 (95%) at 3 months and 267 (96%) at 6 months CA. Physiological consequences of respiratory events separated by event type (apneas, sequential apneas and periodic breathing) and respiratory events combined at 32–36 weeks PMA, 36–40 weeks PMA, 3- and 6-months CA are presented in Tables 2 and 3. Falls in HR during respiratory events increased with increasing age. In contrast, the falls in SpO_2_ decreased with increasing age, with falls in SpO_2_ at 3- and 6-months CA being significantly less when compared to falls at 32–36 weeks PMA (Tables 2 and 3). Falls in TOI did not change with age for any respiratory event type. At all ages, the median group-averaged time spent with SpO_2_ < 90% was <1% TST. However, there was significant individual variation between infants with one infant spending 10% TST with SpO_2_ < 90% 32–36 weeks PMA and another 8% TST with SpO_2_ < 90% at 3 months CA. Similarly, the median group-averaged time spent with TOI < 55% was ≤1% TST at all ages studied, with the majority of this time being during periodic breathing. Some individuals spent significant amounts of time with TOI < 55%: one spent 6% TST at 32–36 weeks, another infant spent 10% TST at 36–40 weeks PMA and another spent 7% TST at 6 months CA.

**Table 2:** Physiological consequences of isolated apneas and sequential apneas at 32–36 weeks PMA, 36–40 weeks PMA, 3- and 6-months CA. Data presented as median [IQR] or *N* (%).

	32–36 weeks PMA	36–40 weeks PMA	3 months CA	6 months CA
Isolated Apneas				
Nadir % change in HR	−7.9 [−10.0, −5.3]	−9.5 [−11.2, −8.1]	−13.1 [−15.5, −11.9] ^a,b^	−16.3 [−18.1, −10.8] ^a,b^
Nadir % change in SpO_2_	−4.5 [−5.5, −2.9]	−2.6 [−5.0, −2.3]	−1.9 [−2.7, −1.4] ^a,b^	−1.7 [−3.1, −1.2] ^a,b^
Nadir % change in TOI	−2.6 [−3.5, −1.7]	−2.5 [−3.5, −1.5]	−2.3 [−3.2, −1.4]	−2.0 [−3.3, −1.3]
*N* spent time in SpO_2_ < 90% (%)	25 (96)	12 (50)	5 (32) ^a^	4 (17) ^a,b^
% TST with SpO_2_ < 90%	0.2 [0.1, 0.6]	0.1 [0.0, 0.4]	0.0 [0.0, 1.9]	0.1 [0.0, 0.1]
*N* spent time in TOI < 55% (%)	8 (31)	6 (25)	4 (21)	0 (0) ^a,b^
% TST with TOI < 55%	0.2 [0.1, 1.8]	0.7 [0.1, 1.7]	0.5 [0.4, 1.2]	-
Sequential apneas				
Nadir % change in HR	−10.0 [−13.0, −7.4]	−10.8 [−12.9, −7.9]	−16.1 [−18.9, −14.5] ^a,b^	−15.2 [−19.7, −12.9] ^a,b^
Nadir % change in SpO_2_	−5.8 [−8.0, −4.2]	−3.6 [−6.6, −2.3]	−2.5 [−4.1, −1.9] ^a^	−4.2 [−4.4, −2.8]
Nadir % change in TOI	−3.5 [−4.6, −2.4]	−2.5 [−4.0, −1.8]	−2.5 [−3.9, −1.5]	−2.4 [−5.3, −1.3]
*N* spent time in SpO_2_ < 90% (%)	22 (88)	13 (54)	1 (8) ^a,b^	3 (30) ^a^
% TST with SpO_2_ < 90%	0.2 [0.1, 0.4]	0.2 [0.1, 0.4]	-	0.3 [0.1, 0.4]
*N* spent time in TOI < 55% (%)	7 (28)	7 (29)	2 (17)	1 (10)
% TST with TOI < 55%	0.1 [0.0, 0.6]	0.2 [0.0, 0.6]	0.5 [0.5, 0.6]	-

*PMA* postmenstrual age, *CA* corrected age, *HR* heart rate, *SpO*_*2*_ peripheral arterial oxygen saturation, *TOI* tissue oxygenation index, *TST* total sleep time

^a^*p* < 0.05 compared to Study 1, ^b^*p* < 0.05 compared to Study 2.

**Table 3:** Physiological consequences of periodic breathing and all respiratory events combined at 32–36 weeks PMA, 36–40 weeks PMA, 3- and 6-months CA. Data presented as median [IQR] or *N* (%).

	32–36 weeks PMA	36–40 weeks PMA	3 months CA	6 months CA
Periodic breathing				
Nadir % change in HR	−12.9 [−18.7, −9.4]	−11.6 [−14.4, −10.7]	−16.6 [−25.9, −13.9]	−17.8 [−21.5, −12.4]
Nadir % change in SpO_2_	−9.0 [−12.4, −7.6]	−8.0 [−9.5, −5.9]	−3.7 [−6.8, −2.4] ^a^	−3.0 [−5.4, −2.0] ^a,b^
Nadir % change in TOI	−4.8 [−8.8, −3.2]	−4.4 [−6.4, −3.2]	−3.6 [−7.5, −1.2]	−2.8 [−4.1, −1.9]
*N* spent time in SpO_2_ < 90% (%)	21 (84)	19 (83)	2 (20) ^a,b^	3 (33) ^a,b^
% TST with SpO_2_ < 90%	0.8 [0.5, 1.4]	0.2 [0.1, 0.9]	1.1 [0.0, 2.2]	0.1 [0.1, 1.4]
*N* spent time in TOI < 55% (%)	10 (40)	8 (35)	2 (20)	1 (11)
% TST with TOI < 55%	0.1 [0.0, 0.6]	1.0 [0.1, 2.1]	0.8 [0.6, 0.9]	-
Combined respiratory events				
Nadir % change in HR	−10.4 [−12.3, −7.3]	−10.6 [−12.5, −9.3]	−13.3 [−16.0, −12.5] ^a,b^	−16.0 [−18.7, −11.1] ^a,b^
Nadir % change in SpO_2_	−6.6 [−7.8, −4.3]	−4.3 [−6.8, −2.8]	−2.2 [−3.7, −1.6] ^a,b^	−2.3 [−3.4, −1.3] ^a,b^
Nadir % change in TOI	−3.7 [−4.8, −2.4]	−3.2 [−4.1, −2.5]	−2.6 [−3.2, −1.5]	−2.1 [−3.3, −1.5]
*N* spent time in SpO_2_ < 90% (%)	26 (100)	21 (88)	5 (26) ^a,b^	5 (21) ^a,b^
% TST with SpO_2_ < 90%	0.9 [0.5, 1.9]	0.3 [0.1, 1.4]	0.1 [0.0, 4.4]	0.7 [0.1, 1.8]
*N* spent time in TOI < 55% (%)	14 (54)	10 (42)	6 (32)	1 (4) ^a,b^
% TST with TOI < 55%	0.1 [0.1, 0.8]	0.7 [0.0, 3.1]	0.5 [0.4, 2.8]	-

*PMA* postmenstrual age, *CA* corrected age, *HR* heart rate, *SpO*_*2*_ peripheral arterial oxygen saturation, *TOI* tissue oxygenation index, *TST* total sleep time

^a^*p* < 0.05 compared to Study 1, ^b^*p* < 0.05 compared to Study 2.

### Relationship between respiratory events and BSID-III outcomes at 6 months CA

Infants completed the BSID-III assessments at a median [IQR] of 6.7 [6.1, 11.4] months CA. Infants had a median [IQR] cognitive composite score of 100 [95, 109], language composite score of 100 [92, 108], motor composite score of 99 [92, 107], social emotional composite score of 100 [95, 115], and adaptive behavior score of 103 [96, 112]. All infants scored >85 in the cognition and adaptive behavior domains. One infant (4%) scored <85 in the language domain, 4 infants (15%) scored <85 in the motor domain, and 3 infants (12%) scored <85 in the social emotional domain.

Stepwise linear regression identified that GA, sex and birth weight were not predictive of BDSI-III scores. %TST with all respiratory events combined and %TSTPB at term CA (36–40 weeks PMA) were significant predictors of language (%TSTevents, Unstandardized β = −0.416, *R*^2^ = 0.165, *p* = 0.039; TSTPB, Unstandardized β = −0.431, *R*^2^ = 0.162, *p* = 0.041) and motor outcomes (TSTevents, Unstandardized β = −0.485, *R*^2^ = 0.180, *p* = 0.031; TSTPB, Unstandardized β = −0.522, *R*^2^ = 0.191, *p* = 0.025), at 6 months CA. %TSTevents and %TSTPB at 32–36 weeks PMA, 3 and 6 months were not associated with any of the BSID-III domains. %TST in isolated apnea or SA were not associated with any of the BSID-III domains. There was no association between nadir % change in SpO_2_ or TOI during respiratory events (isolated or combined) and the BSID-III outcomes.

### Relationship between respiratory events and infant temperament outcomes at 6 months CA

IBQ-R questionnaires were available for 25/26 infants and were completed at a median [IQR] age of 6.4 [6.2, 6.7] months CA. Infants scored (mean ± SD) 4 ± 1.1 for activity level, 3.9 ± 0.9 for distress to limitations, 3.2 ± 1.3 for fear, 3.8 ± 1.1 for duration of orienting, 4.6 ± 1.1 for smiling and laughter, 6.0 ± 0.8 for high pleasure, 5.2 ± 0.9 for low pleasure, 5.4 ± 0.8 for soothability, 5.0 ± 1.1 for falling reactivity, 5.5 ± 0.8 for cuddliness, 4.1 ± 1.6 for perceptual sensitivity, 3.6 ± 0.8 for sadness, 5.3 ± 1.0 for approach and 5.0 ± 1.0 for vocal reactivity. Increased %TSTevents at 32–36 weeks PMA predicted a lower score for perceptual sensitivity (Unstandardized β = −0.046, *R*^2^ = 0.238, *p* = 0.016) and approach (Unstandardized β = −0.032, *R*^2^ = 0.312, *p* = 0.004). Increased %TSTevents at 36–40 weeks PMA predicted a lower perceptual sensitivity score (Unstandardized β = −0.065, *R*^2^ = 0.184, *p* = 0.037) and increased %TSTevents at 6 months CA predicted a higher falling reactivity score (Unstandardized β = 0.073, *R*^2^ = 0.185, *p* = 0.018) and duration of orienting (Unstandardized β = 0.065, *R*^2^ = 0.176, *p* = 0.037).

## Discussion

To our knowledge, this is the first study to investigate the relationship between time spent having short isolated apneas, sequential apneas, periodic breathing and combined respiratory events longitudinally, with developmental and behavioral outcomes in very preterm infants at 6 months CA. As expected, respiratory instability decreased with increasing PNA, however this was extremely variable between infants. We identified that all types of respiratory events were associated with falls in heart rate, cerebral and peripheral oxygenation. %TST in respiratory events, and specifically in periodic breathing at 36–40 PMA, were negatively associated with language and motor scores at 6 months CA. Sleep time spent in respiratory events also predicted decrements in developmental performance on measures of temperament, including perceptual sensitivity, approach, falling reactivity and duration of orienting. Our findings suggest that repetitive desaturation and re-saturation as a consequence of respiratory instability, particularly during periodic breathing, contributes to adverse neurodevelopmental outcomes in preterm infants.

Preterm born infants are at a higher risk for adverse neurodevelopmental outcomes at 2 years of age [28], school age and during adolescence [29, 30] compared to infants born at term. Studies examining prolonged apnea, as occurs during apnea of prematurity, have suggested that the resultant hypoxia is associated with an increased risk for neurodevelopmental impairment [5, 31]. Hypoxia during critical periods of brain formation is associated with detrimental effects on cognitive function, brain adaptive potential and plasticity [13]. In animal studies, rat pups exposed to mild intermittent hypoxia in a pattern similar to that experienced by preterm infants during periodic breathing showed alterations in brain structure and metabolism, as well as systemic and brain inflammation [14]. In addition, intermittent hypoxia (SaO_2_ < 85%) with a mean duration of 2.26 ± 0.11 minutes was associated with permanent neurofunctional deficit and white matter hypomyelination [15]. Furthermore, a study investigating respiratory events in preterm infants born between 24–32 weeks GA and studied at a post‐natal age of 14.5 (range 3–29) days at a corrected gestation of 30 (28–33) weeks found that isolated apneas as short as 5–9 s contributed to overall hypoxemic burden, with even apneas as short as 3 s being associated with hypoxemia [9]. Additionally, the authors found that these short apneas, when clustered in periodic breathing were associated with substantial hypoxemia and bradycardia [9]. Taken together, intermittent hypoxia as a result of respiratory instability has the potential to contribute to adverse neurodevelopmental outcomes in preterm infants.

In our cohort of clinically stable preterm infants, an increased time spent with respiratory events at 36–40 weeks PMA was a determinant of reduced language and motor scores at 6 months CA. Our results suggest that a delayed maturation of respiratory control resulting in respiratory events, particularly periodic breathing, continuing after discharge from the neonatal unit at around term CA may have deleterious effects on neurodevelopment. This suggestion is supported by a study in very low birth weight infants [32], where the authors reported that a delay in resolution of apnea and bradycardia beyond 36 weeks CA was associated with a higher incidence of adverse mental and psychomotor BSID-II scores (2 SD from the mean of 100, <69) at 13 months CA. This suggests there may be a relationship between prolonged respiratory instability to around term equivalent age and adverse neurodevelopmental outcomes. Although both periodic breathing and adverse developmental outcomes may be manifestations of an underlying neuropathology, the exposure to even mild forms of repetitive hypoxia we have demonstrated raises the possibility of a potentially modifiable impact on neurodevelopmental outcome that could be demonstrated via a randomized controlled trial of treatment of periodic breathing.

Among the respiratory events we observed, periodic breathing occupied the highest proportion of the infants’ sleep time, hence, our finding that time spent in periodic breathing, and not time spent in isolated or sequential apneas, was associated with reduced language and motor scores. This finding suggests that intermittent breathing pauses accompanied by mild hypoxia may contribute to poorer neurodevelopmental outcomes in these infants. This finding complements another study in infants born at 27–34 weeks GA and studied at 33–37 weeks PMA, similar to the infants in our cohort, that also found that periodic breathing was the most common cause of prolonged desaturation (SpO_2_ ≤ 80% for ≥4 s) [33]. Furthermore, data from studies in neonatal rat pups has shown that mild intermittent hypoxia episodes (SpO_2_ < 90% for ~2 min), similar to those experienced by preterm infants, was associated with systemic and brain inflammation, altered brain structure and metabolism [14]. A retrospective analysis of continuous pulse oximetry values (SpO_2_) in extremely preterm infants from the Canadian Oxygen Trial (COT) found a significant association between exposure to prolonged hypoxemic episodes (SpO_2_ < 80% for at least 1 min) during the first 2–3 months after birth and the risk of late death or disability at 18 months corrected age CA [34]. A more recent analysis of the COT data has reported prolonged intermittent hypoxemia beginning in the first week after birth was associated with an increased risk of developing severe bronchopulmonary dysplasia (BPD), which is associated with high rates of developmental disability [35].

In our study, not all infants who had respiratory events experienced hypoxia defined as SpO_2_ < 90%. Therefore, the desaturation and re-saturation as a result of recurrent apneas, rather than the time spent with lower SpO_2_, are most likely contributing to the adverse neurodevelopmental outcomes we observed. This suggestion is supported by a comprehensive review by Martin and colleagues [34]. They suggested that episodic hypoxia-re-oxygenation contributes to a proinflammatory cascade that impairs the trajectory of ventilatory control maturation in preterm infants, as has been shown in neonatal rat pups [36].

Importantly, we also measured cerebral oxygenation directly in this study. The nadir % change in TOI for each respiratory type and for all respiratory events combined did not change with increasing PNA, however the number of infants who had falls in TOI below 55% during combined respiratory events decreased with increasing PMA (54% at 32–36 weeks, 42% at 36–40 weeks, 32% at 3 months and 4% at 6 months). In addition, the median nadir % change in TOI for each type of respiratory event was <10%, a change in cerebral oxygenation that would not be deemed clinically significant [37]. However, we and others have previously shown that despite even smaller falls in TOI of around 2% in children with sleep disordered breathing, this condition is associated with impaired behavior and neurocognition [38, 39]. These studies in children further support our contention that the fluctuations in cerebral oxygenation, rather than the degree of hypoxia as a result of respiratory events, likely underpins the adverse developmental outcomes associated with respiratory instability. Cerebral oxygenation <55% has been associated with adverse neurodevelopmental outcomes in preterm infants [27]. In our study not all infants desaturated to a TOI level below 55% during respiratory events. However, it is important to note that this was variable between infants with one infant spending a total of 10% TST < 55% TOI at 36–40 weeks PMA and another spending 7% TST < 55% TOI at 6 months CA during respiratory events. Neither of these infants however had scores <85 in any of the BSID-III domains. The underlying causes of this variability between infants and physiological changes during respiratory events should be further explored.

In addition to developmental outcomes, we also identified that %TSTevents (independent of GA, birth weight and sex) was a significant predictor of several areas of temperament using the IBQ-R. As the IBQ-R is not designed for use in diagnosis of psychological disorders or adverse outcomes, there are no reference scores or thresholds for adverse outcomes. However, infant temperament has been associated with neurodevelopment in preterm infants at 2 years of age [16] and in young children during childhood [40]. Increased %TST having respiratory events at younger ages (32–36 and 36–40 weeks PMA) were associated with lower scores for mean approach and perceptual sensitivity. Decreased scores in pleasure and perceptual sensitivity have been shown previously in very preterm infants with complications such as gray matter abnormalities [41] and high grade intraventricular hemorrhage [42]. Although, none of the infants in our study was diagnosed with intrauterine growth restriction, major congenital and intracranial abnormality, significant intraventricular hemorrhage (Grade III or IV), or a hemodynamically significant patent ductus arteriosus, these complications are associated with adverse neurodevelopment outcomes in preterm infants. Furthermore, a previous study found that positive anticipation, as measured by the approach variable, is associated with increased locomotion ability [43]. Our finding of a lower approach scores taken together with poorer motor scores observed from the BSID-III assessments suggest that infants who spent more time with respiratory events are at a higher risk for motor impairments.

Our study has several limitations that need to be considered when interpreting our findings. Firstly, our sample size was limited to 26 infants. However, our longitudinal design allowed us to follow individual infants across the first 6 months of life, providing longitudinal data on the frequency and physiological consequences of respiratory instability. Furthermore, our findings of significant differences in developmental outcomes are similar to previous nutritional and environmental intervention studies which have shown that a difference of 4–5 points on the BSID-III has clinically meaningful outcomes [44], and this difference has been considered a clinically significant improvement after dietary intervention studies [45]*.* In addition, our study was affected by COVID-19 restrictions, resulting in missing values at 36–40 weeks PMA (8%) and at 3 months CA (27%). Our statistical approach accounted for these missing values when assessing on the effects of respiratory events on BSID-III and IBQ-R outcomes. We assessed the children at 6 months of age to coincide with the final sleep study, which was timed to coincide with the resolution or near resolution of periodic breathing [10, 23]. The BSID-III is suitable for the assessment of infants from 1–42 months, but most studies use it in children over 18 months of age. However, previous studies have shown that assessment at 6 months is reliable in both term and preterm infants for identifying neurocognitive and physical development that are present from 6 months of age and persist to beyond school age [46]. Results at 6 months are predictive of scores at 18 and 36 months [47] as well as at 24 months [48]. We plan an additional assessment at 2 years corrected age and it will be important to identify if our findings at 6 months are confirmed. We also acknowledge that, particularly at the later studies, recording time during sleep studies was shorter than we had planned. This may have impacted on the estimation of respiratory events compared to overnight sleep, leading to either an over or underestimation at each age. However, the amount of time spent with respiratory instability in this study was similar to that reported previously in overnight studies [49] and in previously published studies assessing the frequency of periodic breathing in preterm infants at 35 weeks PMA before hospital discharge [50] and longitudinally after hospital discharge [10]. A strength of our analysis was that we identified both isolated and clustered respiratory pauses and their consequences on cerebral and peripheral oxygenation and their relationship to neurodevelopmental and behavioral outcomes in preterm infants.

## Conclusions

In conclusion, our study identified that the majority of time spent with respiratory events was spent in periodic breathing, and both %TSTPB and %TSTevents at 36–40 weeks were associated with poorer language and motor outcomes at 6 months CA in clinically stable preterm infants who had been discharged home with no concerns of respiratory instability. These short apneas are generally not of sufficient duration to cause bradycardia or desaturation that would trigger monitor alarms, and therefore unlikely to be recognized and treated in the neonatal unit. Our findings add to a growing literature suggesting periodic breathing is not benign and the accompanying brief desaturation and re-saturation may contribute to adverse outcomes in preterm infants. Our results highlight the need for a randomized controlled trial of treatment of respiratory instability identified prior to term equivalent age and potentially continuing until the resolution of periodic breathing.

## Data Availability

The datasets generated and/or analyzed during the current study are available from the corresponding author on reasonable request.
